# Reconciling employment with caring for a husband with an advanced illness

**DOI:** 10.1186/1472-6963-9-216

**Published:** 2009-11-25

**Authors:** Marjolein Gysels, Irene J Higginson

**Affiliations:** 1 King's College London, Department of Palliative Care Policy and Rehabilitation, School of Medicine, London, UK; 2 Barcelona Centre for International Health Research (CRESIB), Barcelona, Spain

## Abstract

**Background:**

Little is known about combining work with caring for a person with advanced illness. This is important given the increasing number of women in the workforce and current policy seeking to increase care in the community. The aim of this paper was to explore the meaning of work for women caring for a husband with an advanced illness and the consequences of combining these two roles.

**Methods:**

A purposive sample of 15 carers was recruited from a hospital and from the community, via the patients they cared for. Their illnesses included chronic obstructive pulmonary disease, cancer, motor neurone disease, and heart failure. Data were collected through semi-structured, in-depth interviews that were tape-recorded and transcribed verbatim. A Grounded Theory approach was used and case studies were developed. NVivo software facilitated the management and analysis of the data.

**Results:**

Caring presented challenges to carers' work life. It diminished productivity or the quality of work, and led to missed opportunities for promotion. Work had an effect on the quality of care and the relationship with the patient, which eventually led to work being given up for caring. Three carers resisted the pressures to give up work and used it as a coping strategy.

**Conclusion:**

A positive choice to remain in employment does not necessarily signal reluctance to care. Caring arrangements need to be understood from the common and separate interests of carers and the people they support.

## Background

There are numerous studies documenting that the majority of patients with life-limiting illness wish to be cared for at home [1]. These studies support the recent changes in policy aiming to base care for people with long-term conditions increasingly in community settings, including the home, and to avoid unnecessary admissions to hospital [2]. However, home care is dependent on several factors, among which strong family support and a good home care program are the most important [1].

Care at home for a patient with advanced disease depends on the availability of an informal carer who agrees to take on the carer role because of her or his relationship with the patient [3]. In most of the literature their status goes unquestioned and their acceptance of this role is assumed. It is clear, however, that taking on the caring role does not necessarily imply a well-considered decision, and carers often claim that they did not have a choice. Due to factors that fall out of their control, there are limited opportunities for carers to exercise choice over services [4-7]. Informal carers' choices may also be restrained by formal carers' attitudes to them [6,8], by the financial implications of formal assistance [9], by subjective factors related to relationships [6,9,10] or gender roles [6]. Carers' views and preferences are less well represented in the literature, particularly those exhibiting resistance to their role [1,11]. This may be because caring for a sick relative is associated with deeply held moral expectations, especially for women who are socialised into a caring role [12]. As a result, unwillingness to care remains hidden [11]. Focusing on alternative choices runs counter the interests of a system relying on informal carers for the support of people with a long-term illness.

Research on carers has most often used the concept of burden, which implies the negative impact of caring on carers' own health and well-being [13-15]. From these studies we know that carers are vulnerable to negative physical, psychological and social consequences. Caring is demanding, especially when this comes on top of other responsibilities such as work. Employment for women is increasing and this means that both men and women will combine these roles [13,14]. Most carers succeed in balancing work and care [16], but the evidence indicates the negative effect work can have on carers. Balancing the two responsibilities often requires meeting conflicting demands which can cause considerable stress. Reconciling these roles can become very difficult, especially in the advanced stages of illness. After having exhausted alternative arrangements by reducing or adjusting working hours, many carers eventually give up work [17].

Due to the considerable contribution of informal carers to the health care system, and in the context of an ageing society, some policy documents have recognised the needs of carers. In the context of the UK's Choice programme, the government set out to improve working conditions to enable carers to continue caring or return to work [18]. A study looking at the extent to which the needs of employed people with caring responsibilities were supported in the workplace found that most of the organisations studied were able to provide appropriate support for carers, such as leave policies, carer-friendly working arrangements, access to a telephone, and supportive line-managers and co-workers [8]. These initiatives were based on evidence showing that work is beneficial to carers; it offers them financial independence, assists in covering costs of care, provides a break from caring, and allows social relations and satisfaction with achievements [17,19,20].

Little is known about combining work with caring [21]. This needs to be understood against the background of a larger number of women in the workforce and the current policy orientation to care for those affected by long-term illness. We also know little about how carers feel about work and caring. Caring can impact negatively on work, but what is the effect of work on caring? Care giving is a complex social process which develops from commitments in personal relationships. We need empirical evidence to understand how choices are made and negotiated between carers and patients. We know that at a certain point, most carers will sacrifice work for caring, but it is not clear why some people choose to carry on working in spite of the double burden. When do the advantages of employment start to outweigh the disadvantages? Does a positive choice for work signal reluctance to care? With this study we aim to explore the meaning of work for women caring for a husband with an advanced illness and the consequences of combining these two roles.

## Methods

Data were collected in the context of a qualitative study exploring the experiences of breathlessness among patients with advanced lung cancer, chronic obstructive pulmonary disease (COPD), cardiac failure and motor neurone disease (MND), at different stages of their illness, and in different care settings. The inclusion criterion was broad - patients experiencing breathlessness as compromising their quality of life - to allow for a wide range of experiences. Carers who were accessible and willing to participate were also interviewed. The study took place in a large teaching hospital and in the surrounding community in London, between July 2005 and March 2006.

Patients were recruited during visits to respiratory clinics at the hospital, at specialist respiratory nurses' ward rounds and consultations, through "Breathe Easy" service users meetings, and from the disease registers of the primary care team in a general practitioner's surgery. Patients' information was retrieved from patients' records or specialist databases and approval for this was part of the main ethics application (see below). Patients were then asked whether they agreed that we approached their carers for an interview and if they thought their carers would be willing to take part in the study. Demographic data and other information were obtained directly from carers during the interviews (See Table 1). This paper is based on a purposive sample of 15 informal wife-carers who provided care for their husbands with advanced disease at home.

**Table 1:** Carers details

Recruitment setting	Types of patients	Number of wife carers interviewed	Number of times interviewed	Age of wife carers	Wives with no issues around caring and work	Wives having issues with care and work
Outpatient clinic	Patients with cancer	1	1 time	61	1 housewife	
	
	Patients with COPD	2	1 time	46 72	1 housewife	1 gave up work
	
	Patients with cardiac failure	3	1 time	58 59 61	1 housewife	2 combining work and caring
	
	MND patients	8	1 time	Range: 40-72 Median: 57	1 volunteer work 1 employed 4 housewives	2 combining work and caring

Community	Patients receiving palliative care	1	3 times	59	/	1 employed

Totals		15			9	6

Consenting carers were interviewed at home, except for two who preferred to be interviewed in the researcher's office. The interviews were open, in-depth and semi-structured[22] and lasted between 40 and 150 minutes. They were exploratory in the sense that they allowed respondents to discuss on any topic relevant to them, but a topic guide was used to ensure that all the necessary topics were covered. Topics included: family background, current daily life situation, attitudes and responses from the social environment, views on services, and attitudes to the future. All interviews were conducted by MG, one of the authors of the study, they were tape recorded and transcribed verbatim by MG and the research assistant. Field notes were kept in situations where it was inappropriate to tape record.

Interpretation of the results was from a Grounded Theory perspective [23,24], which aims to discover hypotheses about a phenomenon, in this case balancing work with care giving, by grounding them in empirical data, especially in practical interactions and social processes. This was facilitated by the use of the computer software package Nvivo, which is designed to assist in the management and analysis of qualitative data. Through the constant comparative method[23] incidents, events and activities were identified and constantly compared to emerging categories. Practically this involved reading and coding the transcribed texts of interviews and discussions in several stages. Sections of texts relating to particular topics or themes were labelled and related to each other, giving rise to more abstract generalisations. Case studies [25,26] were developed to analyse the flow of events that led to carers' decisions regarding caring and work without losing sight of influencing factors.

In the context of the broader programme on breathlessness, a team of researchers worked on related issues of the symptom, which facilitated the exchange of experiences, the comparison of data and further discussion, thus contributing to the verification of interpretations.

Ethics approval was granted by the King's College Hospital Local Research Ethics Committee and by representative bodies (the hospital and community research and development committees) in the boroughs in which the study was carried out. Data were stored anonymously and pseudonyms are used throughout this paper.

## Results

For demographic data and other information of the sample of carers on which this paper is based see Table 1.

**Figure 1:**
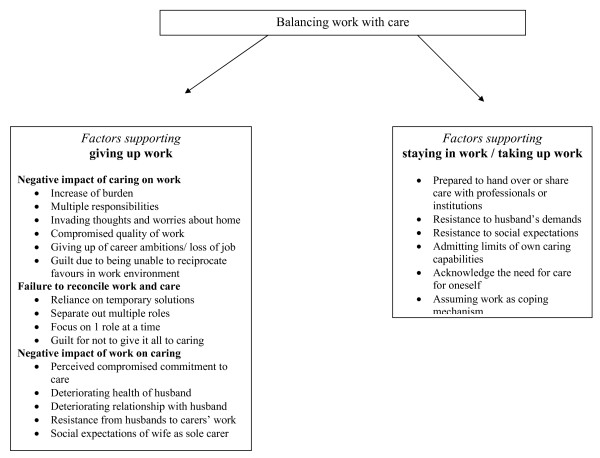
**Factors influencing work and care**.

There were two categories of responses from carers who were employed while caring for a husband with advanced disease. There were those who would eventually give up their job to care full-time for their husbands at home, and there were those who would resist the pressures (from husbands, from their work context, or their social environment) to give up work, or who took up work while caring. These categories are composed of different dimensions that determined decisions made regarding work and caring, the benefits and challenges associated with either work or caring, or the combination of both, and they either facilitated or impeded work or care (see Figure 1). Actions and reactions towards caring and work are processes that develop over time and in the context of relationships and wider conditions.

### Carers who eventually gave up work for caring

#### Impact of caring on work

The carers in this study mentioned work as one of the aspects they were concerned about while caring for their husbands. Caring threatened work, either the quality of it or the sheer possibility of keeping their job.

"...things have got to such a state that I find it very hard to concentrate, and I'm meant to be meticulous about spotting things and I don't feel I [...] can do that, I've also got to be creative and I don't feel very creative. " (carer of a cancer patient)

All carers said that their employers were sympathetic towards their caring commitment and they appreciated their understanding and the flexibility that allowed them to carry out their caring duties. This created a feeling of reciprocal commitment towards their employers. They wanted to give something in return for their employers' kindness. When they felt they could not, they started feeling as if they were taking advantage of the situation and this lead to feelings of guilt. For some this was unacceptable and it led them to give up work altogether.

The carers felt they were taking on enormous responsibilities, as they were looking after their husbands, had professional duties, managed a household, and sometimes carried responsibility for other dependents. They described how they never stopped their activities and how tired they were at the end of the day.

Caring impacted on their working lives as they carried their worries with them throughout the day. They kept in regular contact with their husbands at home. Sometimes they went to work while leaving their husbands who were not feeling well.

*"...but [...] if I come to work and I know he's been particularly unwell it niggles me all day long..." *(carer of heart failure patient)

Caring interfered in such a way that they missed opportunities to develop in their career.

*"I've now had to cut back on it and I turned down a promotion" *(carer of heart failure patient)

#### Reconciling two responsibilities

Some carers said that they were able to go to work because their husband had professional help in their absence, or there were people who kept an eye on him during the day.

A strategy carers mentioned in order to manage the two roles, was to concentrate on the task at hand when at work and bracket out their worries about care giving.

*" [...] I have to almost metamorphosise to take off carers hat, put work hat on, because otherwise I wouldn't survive in the job." *(carer of heart failure patient)

In some cases patients objected to this situation, asking for more companionship. Those carers who were most successful in maintaining their jobs resisted their husbands' dissatisfaction.

The interviews showed that when illness struck, it had consequences for the usual arrangements in a household, priorities shifted, and plans were reconsidered. In acute situations these rearrangements were made as it best fitted the circumstances. There was little opportunity to reflect on what was happening and things took their course for the time being. Elisabeth described how she took over as breadwinner from her husband when he could not return to work after he had a heart attack. Initially she worked part-time to be able to take care of him and her son and she was able to rely on her sister to help out when extra help was needed. When the rearrangements seemed to work, Elisabeth started working full-time. These were developments where there was little choice involved.

Decisions to become a full-time carer developed over time and in the context of a relationship with the patient. Elisabeth had grown into her work, since she took over the role of breadwinner from her husband. She described the dread with which she anticipated the necessity of reducing working hours due to her husband's deteriorating health. Reflections such as these balance options and determine future life directions and commitments.

*"I think [...] after this last episode the emphasis has changed and I see myself going into the caring role. That's it, [...] I did sort of start discussing [...] working part time, [...]I've managed to persuade them that I need to be working at home one day a week, which is what I'm doing at the moment. So [...] the balance is shifting." *(carer of heart failure patient)

#### Impact of work on caring

The decision to give up work was not easy for the carers and it went together with ambivalent feelings. Carers took this step after a long period of consideration, and first trying to make it work both ways. The circumstances that drove carers to give up work were the poor physical and mental state of their husbands. One carer attributed her judgement about the necessity to reduce her working life to the increasing emotional dependency of her husband.

*"... something may happen, I'm suddenly called into a long meeting which I expected to last an hour and it ends up lasting three hours and I haven't been able to tell him, then he's quite stressed about that." *(carer of heart failure patient)

One carer explained that the main reason for leaving work was her husband's loneliness when he was left at home.

*"...but sadly I had to leave that (work) in January because [...] it was becoming obvious that Joe needed more intense care and we weren't gonna get any progression with me at work full time and him on his own." *(carer of COPD patient)

The decision to leave work was motivated by the carers' perception that work had a negative influence on their husbands' health or well-being. Some carers felt that they had lost a part of the commitment they found they should put into caring, which now went into paid work. This led to feelings of guilt.

*"I try not to get guilty about it, because then you think, oh god, I should have stopped work 6 months ago, a year ago! And you daren't even think about that because you can't, you know, take that on to deal with now." *(carer of COPD patient)

Leaving work for caring was seen as an investment into their husbands' well-being. When Cathy had taken the decision to give up her work to commit herself to caring for her husband, she found purpose in caring. She said that her husband's health was deteriorating and that her input was needed to change his lifestyle. Without Cathy, he did not bother to eat and did not persevere in giving up smoking.

*"I'm sure he's had the odd (cigarette), he's probably gone to our neighbour and said: 'Oh give me a cigarette for goodness sake'. [...] but I don't think he's had more than a couple because, I have threatened that if, [...] he smokes, I'll give up [...]. And you do get angry and I've said there's no point in me being at home and giving up work and, if you're just gonna continue, it's a waste both of our time." *(carer of COPD patient)

Giving up work for caring was motivated by the uncertainty of a progressive illness and the carer's wish to do everything she can for her husband. At the same time the end of a job also meant a lost world.

### Those who continued to work or took up work

There were three carers who remained in paid work despite the circumstances that would usually motivate carers to leave employment.

#### CASE 1

Lynn had always been the main breadwinner; she worked as an editor of a well-read magazine. She described how she enjoyed her work, which was at times very demanding. Over the years her husband, Richard, had gradually become ill. He had COPD and developed severe breathing problems and four years earlier he was diagnosed with lung cancer. For the last years he received care at home and he was seen by the palliative care team of the hospital in the area. He enjoyed going to the day care centre of the hospice in the neighbourhood. This allowed Lynn to continue working. However, Richard's health worsened and he required full-time attendance. When looking for a solution she was informed that Richard already received the maximum care available. When I met them for the first time, the decision had just been made for Richard to go to a care home. In the weekends Lynn cared for him at home.

When asked about giving up work, Lynn responded that this was not the right decision for them:

"Not not not an option because I'm still supporting both my sons, so financially it would be impossible. Also I don't think I would be a very good carer [...] and because I'm emotionally involved I think would make it even harder. And although I want to be supportive to Richard, I just don't think I could be a full-time carer. [...] I think he gets the best from me if I'm allowed to go to work and then I'm very happy to be with him..."

Lynn tried to maintain as much normality as possible in their lives. She had a social life and went out with her friends or colleagues from time to time. She did not speak to them about the problems she faced at home. For this she had a therapist whom she paid for her services.

"...it would be very easy for me not to have any social life at the moment with working full time. And it sometimes feels wrong to deprive Richard of my time when I'm not at work. But I feel, and this is what I talk about with my therapist..., it is very important that [...], because I will have to carry on afterwards when Richard is no longer here... I must keep up friendships and have some form of social life and I think that's important for my mental health."

Lynn described how she and her husband discussed these matters together, but at the same time the difficulty of accepting that their lives took different directions appeared from her story.

" [...] he understands that logically but of course emotionally, [...] he finds it difficult because he would rather I was there all the time."

Richard's illness had affected both their lives and Lynn did what she thought would cause the least damage to their lives. She tried to save their relationship, which had changed with the severity of Richard's illness.

"...so I didn't want our relationship to suffer which I'm sure it would [if I stopped work].

Lynn said they were no longer relating to each other as equals, but she now had to assume responsibility for him as a parent over a child.

"...you then become the sort of parent, [...] and [...] now I'm the carer and I have to look after Richard, our relationship has changed so it's no longer one of equals, which is difficult for him..."

She wanted to be able to care in a sincere way. She thought she would not be capable of caring and feeling happy about it, if it became a full-time task, and she wanted to avoid the pain that would cause.

"... if I have time off and I do things without him, I do find it makes it much easier to be patient and loving and I do things with him [...], for good grace. And I'm not feeling resentful which I think, [...] if I was a full time carer, I would find that very hard."

#### CASE 2

Tricia was married to Jack who had been diagnosed with MND seven years before. Jack had become very disabled. He used a wheelchair and needed assistance with fine movements. He had developed severe breathing problems, which were reasonably well controlled with the "nippy" (non-invasive positive pressure ventilator).

In the interview with Tricia she expressed her appreciation of the strength with which Jack faced his illness, and she described how grateful he always was for her care. She broke down shortly after the interview had started, and while crying she described the difficulties of caring.

"...he seems to cope better than I do. Sometimes I get very tired coz it's from first thing in the morning when I get him up, till of a night when I'm putting his mask on and I'm getting him into bed. Yes, so he would never ever be left alone, well I have to leave him alone to go out coz I do still work at the surgery."

..."but I'm always crying everyday, if a day goes by and I'll not cry it's a miracle and if somebody rings up, [...] just says something, you know, and if people are kind..."

She explained that it was out of the question of leaving her job to care for her husband. Now, after retirement, she continued part-time with her job at the surgery where she had worked as a nurse for 26 years. She explained that this was the only way she could cope with her caring duties at home. Work provided her with a break from her daily worries.

"No that's the last thing I want [to give up work]. I'm well retired and [...] I don't need to go for the money and Jack doesn't want me to give up because it's [...] my only outlet..."

Her work gave her a sense of accomplishment. She used her skills with confidence, and felt she was in control of her tasks.

"...meeting people, [...] really that's all I have other than here. So I want to keep it on as long as I can and they keep me on because erm having been there 26 years and done it all, they call me when they need me... and I love it."

She also applied her nursing skills at home and took pride in the fact that she could make a difference to Jack's life by saving him from having to go to the hospital.

" I don't want him to get MRSA, I dress it myself coz I can do all that, so in lots of ways we're self sufficient we can look after ourselves."

The prospect that Jack's situation was only getting worse and the unpredictability made her despair. Her work was an environment where she had to be strong,

"So it's just a case of coping [...]. And you see, I can't cry at work. I'm talking to the patients all the time and whilst I'm there for three to four hours I'm myself. That's how I cope, so its not that I say I need to go to work, I go coz it's the only thing that keeps me going..."

#### CASE 3

Helga cared for her husband Bert, who was diagnosed three years previously with MND. Bert was practically unable to move his limbs and was restricted to bed. Helga shared the care with her daughter, who was unemployed. This allowed Helga to go out to work. She had a job as a lollypop lady. Her daughter received a carer allowance.

"...so we do it like between us sort of thing so it helps me out, coz you need a little break now and again, don't ya."

Sometimes, caring became too much and that is why she asked for assistance from a professional carer. At first Bert had difficulty accepting this, but Helga explained she needed the extra help. Bert wanted his wife to be with him and carry out these tasks, just as in Cases 1 and 2 above.

"...me daughter explained to him: 'Mum goes to work, she does the shopping, she's looking after the house, she's looking after you, [...], she needs a little bit of extra help to do it', and I think he's just about understanding that now."

Going out to work fulfilled the same function as having respite care for him.

"...that's why I explain to him that when he goes into the hospice, I need that rest to build my batteries up again, otherwise I'll go down."

## Discussion

In this paper we examined the circumstances of carers who combined employment with caring for their husbands who had a long-term, life-limiting illness. We examined interviews with carers who had reflected on this and who had expressed their worries, anticipations, decisions, regrets about fulfilling both roles, the transition from employment to caring, and their decision to hold on to employment. The analysis of these interviews was undertaken to supplement epidemiological research examining relationships between employment and caring, and between work, health and demographic variables. Through a qualitative design we sought to understand how wife carers experienced work in relationship to caring and the varieties of meanings they attributed to these roles.

The interviews showed how caring impacted on work. Competing demands threatened work by diminished productivity, late arrivals or periods of absence [9]. This affected carers' perceptions of self-worth. Although carers reported that their employers were sympathetic to their problems, they felt guilty about the exceptional treatment they received. Not being able to reciprocate was experienced as unacceptable and this played a role in their decisions to give up work altogether. These lines of action resonate with the common understanding of a strict division between work belonging to the public world and the home, and family to the private world.

This public/private notion also appeared in carers' strategy to manage both roles, to fully attend to work and switch off from intruding thoughts from home. This contributed to the time put into work, and it meant less attention for the sick husband who remained at home. In several cases, husbands felt a declining investment in their care. It shows that work arrangements and caring are negotiated between patient and carer, depending on their relationship, confirming previous evidence [27]. They appear as temporary solutions, to serve the needs and goals of the moment, rather than fixed choices, and this also supports the literature on choice, suggesting that this concept and its practice are highly problematic for informal carers [28].

The husbands at home missed their wives' companionship and they started to reclaim this in different ways. Disengagement from the sick husband was one of the effects of work on carers. When the carers started realising this, they felt guilty. This compelled carers to give up work and dedicate themselves full-time to caring.

Losing one's job went together with regret. The investment that carers had made in their work, which in some cases had detracted from the quality of caring, was at the same time experienced as a retreat from the worries and restrictions that could be part of long-term care giving at home. Work and caring appeared in many ways as oppositional. Work fulfilled an important positive role in the carers' lives; it allowed them to preserve their identity, it gave them a broader network of social contacts and a sense of competence and accomplishment. Caring was characterised as insular and constant, without breaks or hope of improvement. Illness affected both the patient's and the carer's life, in that it radically changed life plans. Work offered a way to undo the disruption that long-term illness presented to carers, and appears as one of the strategies that carers can employ to reconstruct parts of their lives [29,30]. This does not mean that carers could not find meaning in caring [31]. Cathy, for example, had given up work to care for her husband and had adopted this as a challenge. However, through work, the other carers obtained an opportunity to rebuild what was lost through illness. Despite these losses, many carers acknowledged positive elements of caring. These insights complement other work on caring, claiming that inclusion of positive experiences rather than only focus on 'burden', is necessary to allow for a full picture of the caring experience [31].

Against this background, we were able to place the three cases of carers who resisted the pressures to give up work. They had adopted work as a coping strategy. When their husbands became increasingly dependent on them, they did not consider leaving work to become a full-time carer as an option. Lynn took the future into account when reflecting on her position: giving up work in the last years of her career would mean the end of her professional life, which would have serious material consequences for her and her sons. She recognised that she would not make a good carer if she had to take on this responsibility full-time. She explained that caring would be detrimental for their relationship. Going to work allowed her to take on her caring role 'for good grace'. Work for Lynn was part of her identity and self-realisation.

In a different way, Tricia also used her work as a coping strategy. She recognised that her vulnerability, which expressed itself with frequent crying, bordered to depression. Her job as a nurse required her to be strong, to support others in a professional way: to provide solutions to others' health problems, and detach again. This distracted her from her own problems at home on which she had little impact.

Helga explained that caring was manageable, as long as she could maintain normality by going out to work. She expressed the need to take breaks from caring in order to recharge and come back with renewed energy. In this way, her job offered her respite from her caring duties.

Does resistance against giving up work signal reluctance to care? It does in the sense that the time spent at work cannot be spent on caring. However, the carers in this sample, held on to work in order to be able to care for their husbands. Lynn recognised her limitations as a carer and saw professional care as a better solution. She wished to give 'good care', which she was able to give when it was not her sole activity. Tricia wanted to realise both her and her husband's wish to provide home care. She could only cope with the distress of his illness by going out to work and concentrate on the daily troubles of others. Helga used her work in a similar way, to give her a break from caring and feel part of daily, normal life.

Reluctance to care goes against social expectations and can evoke negative reactions from the social environment or health professionals [11]. The findings of this paper show that an inclusive approach [6,27] needs to be used in understanding the needs of both parties, the carer, as well as the patient. Joint and separate interests and views need to be considered to avoid adverse consequences and allow for positive outcomes.

## Limitations of this study

From the literature we know that due to the stresses associated with the combination of paid work and caring for a patient at home with advanced disease, carers eventually give up work (10). Due to the small sample size in this in-depth qualitative study it was not possible to determine how exceptional these cases who resisted the pressures to give up work were. Moreover, a longitudinal prospective study using interviews at different time points could have shed light on factors that influence decisions about work and care.

## Conclusion

This study showed that the variations in caring arrangements for a husband with advanced illness, depends on the relationship of the carer with the patient; on family situations, values attached to role expectations and commitments to other responsibilities. These different arrangements were temporary solutions to integrate caring into life with other, competing responsibilities. From the carers' reflections on work and caring we identified some common concerns. The carers spoke about the challenges that caring presented to work life: it diminished their work productivity, led to missed opportunities for promotion and sometimes to giving up work.

The demands of work reduced time spent on caring. Carers' coping strategies to juggle both responsibilities required them to separate both tasks, which had an effect on caring quality and the relationship with the patient. This was the main reason why they eventually sacrificed work for caring, despite their regret to lose the advantages that were associated with work.

The carers who resisted the pressures that forced them to leave employment used work as a coping strategy. The wives' reluctance to dedicate themselves exclusively to caring did not mean they were not willing to care. Their commitment to other roles may be a way to enable them to retaining the caring role. Arrangements regarding caring need to be considered from the common and separate interests of carers and the people they support.

## Competing interests

The authors declare that they have no competing interests.

## Authors' contributions

MG conceived the study. IJH contributed substantially to the design of the study on which these data are based and to all the procedures of gaining ethics permission. MG conducted the data collection. MG drafted the manuscript. IJH revised it critically for important intellectual content. Both authors have read and approved the final version of the manuscript.

## Pre-publication history

The pre-publication history for this paper can be accessed here:

http://www.biomedcentral.com/1472-6963/9/216/prepub
